# Does a lack of vaccine side effects correlate with reduced BNT162b2 mRNA vaccine response among healthcare workers and nursing home residents?

**DOI:** 10.1007/s40520-021-01987-9

**Published:** 2021-10-15

**Authors:** Oladayo A. Oyebanji, Brigid Wilson, Debbie Keresztesy, Lenore Carias, Dennis Wilk, Michael Payne, Htin Aung, Kerri St. Denis, Evan C. Lam, Christopher F. Rowley, Sarah D. Berry, Cheryl M. Cameron, Mark J. Cameron, Kenneth E. Schmader, Alejandro B. Balazs, Christopher L. King, David H. Canaday, Stefan Gravenstein

**Affiliations:** 1 Case Western Reserve University School of Medicine, Cleveland, OH USA; 2 Geriatric Research, Education and Clinical Center, VA Northeast Ohio Healthcare System, Cleveland, OH USA; 3 Ragon Institute of MGH, MIT and Harvard, Cambridge, MA USA; 4 Division of Infectious Diseases, Beth Israel Deaconess Medical Center, Boston, MA USA; 5 Marcus Institute for Aging Research, Hebrew SeniorLife, Boston, MA USA; 6 Geriatric Research, Education and Clinical Center, Durham VA Health Care System and Duke University Medical Center, Durham, NC USA; 7 Division of Geriatrics and Palliative Medicine, Alpert Medical School of Brown University, Providence, RI USA; 8 Center on Innovation in Long-Term Services and Supports, Providence Veterans Administration Medical Center, Providence, RI USA

**Keywords:** COVID-19, Reactogenicity, Immunogenicity, Vaccination, Nursing homes

## Abstract

**Background:**

The BNT162b2 SARS-CoV-2 mRNA vaccination has mitigated the burden of COVID-19 among residents of long-term care facilities considerably, despite being excluded from the vaccine trials. Data on reactogenicity (vaccine side effects) in this population are limited.

**Aims:**

To assess reactogenicity among nursing home (NH) residents. To provide a plausible proxy for predicting vaccine response among this population.

**Methods:**

We enrolled and sampled NH residents and community-dwelling healthcare workers who received the BNT162b2 mRNA vaccine, to assess local or systemic reactogenicity and antibody levels (immunogenicity).

**Results:**

NH residents reported reactions at a much lower frequency and lesser severity than the community-dwelling healthcare workers. These reactions were mild and transient with all subjects experiencing more local than systemic reactions. Based on our reactogenicity and immunogenicity data, we developed a linear regression model predicting log-transformed anti-spike, anti-receptor-binding domain (RBD), and neutralizing titers, with a dichotomous variable indicating the presence or absence of reported reactions which revealed a statistically significant effect, with estimated shifts in log-transformed titers ranging from 0.32 to 0.37 (all *p* < 0.01) indicating greater immunogenicity in subjects with one or more reported reactions of varying severity.

**Discussion:**

With a significantly lower incidence of post-vaccination reactions among NH residents as reported in this study, the BNT162b2 mRNA vaccine appears to be well-tolerated among this vulnerable population. If validated in larger populations, absence of reactogenicity could help guide clinicians in prioritizing vaccine boosters.

**Conclusions:**

Reactogenicity is significantly mild among nursing home residents and overall, subjects who reported post-vaccination reactions developed higher antibody titers.

**Supplementary Information:**

The online version contains supplementary material available at 10.1007/s40520-021-01987-9.

## Introduction

The scourge of the COVID-19 pandemic led efforts to curb its impact, one of which resulted in Pfizer-BioNTech's BNT162b2 SARS-CoV-2 mRNA vaccine and its emergency use authorization [1]. The vaccine and its mRNA technology’s newness have raised safety concerns that factor into its acceptance for some [2–4], despite safety reported in the BNT162b2 mRNA phase 3 trial [5].

The phase 1 BNT162b2 clinical trial reported fewer adverse events (AE) in healthy aged (> 65 years old) than younger subjects [6], but trials leading to its original authorization did not enroll nursing home (NH) residents, leaving a data safety gap [7]. Yet, NHs led the country in mortality for both residents [8] and staff [9], so they were given priority access to the vaccine once it was authorized [10]. Subsequent metadata studies give evidence to the safety and effectiveness of the mRNA vaccines in this vulnerable population [11, 12]. Here, we describe the incidence and pattern of reactions reported by BNT162b2 mRNA vaccine recipients in NH and how this compares with younger community-dwelling healthcare workers as controls, providing real-world data on the vaccine’s reactogenicity profile among this vulnerable population.

Vaccine reactogenicity and antibody response decline with age [13–15]. On the upside, both elderly and younger recipients of the BNT162b2 mRNA vaccine who had a prior SARS-CoV-2 infection have been reported to have higher immune responses than those not previously infected [16, 17], but, on the downside, they experience a corresponding increase in reactions to the vaccine [18, 19]. These synchronous associations between post-vaccination reactions and immune response may have contributed to the wide-held conjecture that greater reactogenicity might signal better immunogenicity [20]. Also, as feverishness to influenza vaccine might predict greater antibody rise [21], we hypothesized that BNT162b2 mRNA vaccine reactogenicity generally correlates with antibody response and thus, further explored a possible relationship between the reactions reported by these vaccine recipients and their immune response to the vaccine.

## Methods

### Study design and population

Our study population draws residents from 4 NHs across Northeast Ohio, USA, and community-dwelling controls that are mostly health care workers. Study approval was obtained from the New England Institutional Review Board. All subjects provided informed consent. Participants were enrolled in the study if they were willing to receive the BNT162b2 mRNA vaccine and able to provide consent themselves. Participants were deemed “*Prior SARS-CoV-2-infected*” if they had a positive PCR or antigen test done prior to enrollment in the study that confirmed acute SARS-CoV-2 infection, and/or high antibody titer to SARS-CoV-2 spike and receptor-binding domain (RBD), and “*SARS-CoV-2-naive*” if otherwise.

### Reactogenicity assessment

Participants were asked to keep a reactogenicity log after receiving each dose. Subjects were educated to record the occurrence of symptoms for the 8 days following receipt of each dose. Solicited Local symptoms include “swelling”, “pain/tenderness at injection site”, “induration”, “redness”, while Systemic symptoms included “headache,” “fatigue,” “fever,” “muscle pains,” “body rash,” “shivering,” “gastrointestinal” (GI) symptoms (including nausea, vomiting, and diarrhea). In addition to the solicited symptoms, subjects were asked to record any other symptoms as “Other.” Participants were instructed to report each symptom with a severity grade score [22] defined as:

- 0-No symptoms
- 1-Mild; Awareness of symptoms, but easily tolerated.
- 2-Moderate; Discomforting enough to cause interference with usual activities.
- 3-Severe; Incapacitating with an inability to work or do usual activities.

Fever was defined as [23]:

- Grade 0: < 38.0 °C
- Grade 1: ≥ 38.0 °C to 38.4 °C
- Grade 2: > 38.4 °C to 38.9 °C
- Grade 3: > 38.9 °C to 40.0 °C

Participants were allowed to provide a subjective assessment of induration, as they were neither provided with a ruler nor educated on objective means of assessment.

A verbal interview was conducted when we collected the symptom log to validate log entry, improve accuracy or to serve as the assessment of reactogenicity if the written log was not completed.

### Immunogenicity assessment

Participants provided pre- and post-vaccination blood samples drawn within 14 days before BNT162b2 mRNA vaccination and within 14 ± 3 days of receiving the 2nd dose, respectively. Immune response to the vaccine was assessed using IgG to spike protein and its receptor-binding domain (RBD) using bead-multiplex immunoassay and serum neutralization titers in a SARS-CoV-2-pseudovirus neutralization assay.

**Anti-spike and anti-RBD assay**. Stabilized full-length S protein (aa 16–1230, with furin site mutated) and RBD (aa 319–541) were conjugated to magnetic microbeads (Luminex) and Magpix assay system (BioRad, Inc), the mean fluorescent index is recorded after antigen-specific IgG is detected in patient serum/plasma using PE-conjugated Donkey F(ab)2 anti-human IgG, with Fcγ (Jackson Immunological). Relative antibody units were then ascribed from an internal standard control convalescent serum as previously described [24].

**SARS-CoV-2 pseudovirus neutralization assay**. To compare the neutralizing activity of vaccine recipients’ sera against coronaviruses, we produced lentiviral particles pseudotyped with vaccine strain spike protein as previously described [25]. Briefly, neutralization assays were performed using a Fluent 780 liquid handler (Tecan) in 384-well plates (Grenier). Three-fold serial dilutions ranging from 1:12 to 1:8,748 were performed and added to 50–250 infectious units of pseudovirus for 1 h. pNT50 values were calculated by taking the inverse of the 50% inhibitory concentration value for all samples with a pseudovirus neutralization value of 80% or higher at the highest concentration of serum.

### Statistical analysis

Differences within the study population between NH and control subjects and those with and without reported reactions were evaluated using Chi-square tests for categorical variables, Wilcoxon rank-sum tests for age, and t tests for anti-Spike, anti-RBD, and neutralizing titers log-transformed values. Differences in reactions to the two doses, overall and within subgroups, were assessed using McNemar’s test for paired dichotomous data. To examine the relationship between reactogenicity and antibody levels, we estimated ordinary least squares linear models predicting log-transformed anti-spike, anti-RBD, and neutralizing titers, controlling for NH vs. control, gender, age, prior SARS-CoV-2 infection, and the interaction of prior SARS-CoV-2 infection and age, based on prior findings [24]. We included a dichotomous variable indicating the presence or absence of any reported reactions. The estimated effect of this variable represents a difference in geometric means between these groups after adjusting for covariates. Differences were considered statistically significant if p < 0.05. Variables not found to be statistically significant were excluded from the final model and graphical summaries of observed values and predicted values were generated based on the final model. All analyses were done using R 4.0.3.

## Results

### Baseline characteristics

We report the reactogenicity data on 193 recipients of the BNT162b2 mRNA vaccine. Of these, 85 reside in NHs (median age 74; range 48–99 years); 51 were SARS-CoV-2-naive at the time of vaccination and 34 had a prior SARS-CoV-2 infection (Table 1). The control group has 108 community-dwelling volunteers (median age 48; range 26–78 years), made up of 33 prior SARS-CoV-2-infected subjects while 75 were SARS-CoV-2-naive. Some subjects in the control group did not have a pre-vaccination blood draw and were determined to be SARS-CoV-2-naive by not having a prior positive PCR or antigen test, and below-threshold anti-Nucleocapsid levels in the post-vaccine blood draw [26]. Despite an age overlap in the NH and control group participants, Chi-square test reports a significant difference in the age distribution (*p* < 0.001). Most of our participants are Caucasian (84%) and were SARS-CoV-2 naive (65%).

**Table 1:** Baseline characteristics and comparison of reactogenicity

	All subjects	NH	Control	NH vs. Control	Reactions	No reaction	Reactions vs. No Reaction
Number of Subjects	193	85	108		125	68	
Age: median (IQR)	61(48,74)	74(68,83)	48(39,56)	< 0.001	53(43,65)	72(66,81)	< 0.001
Age: range	26–99	48–99	26–78		26–92	35–99	
Male	102(53%)	51(60%)	51(47%)	0.105	62(50%)	40(59%)	0.282
Female	91(47%)	34(40%)	57(53%)		63(50%)	28(41%)	
Race: white	163(84%)	74(87%)	89(82%)	0.078	104(83%)	59(87%)	0.582
Race: black	20(10%)	10(12%)	10(9%)		13(10%)	7(10%)	
Race: other	10(5%)	1(1%)	9(8%)		8(6%)	2(3%)	
Prior SARS-CoV-2	67(35%)	34(40%)	33(31%)	0.224	41(33%)	26(38%)	0.649
SARS-CoV-2-Naïve	126(65%)	51(60%)	75(69%)		84(67%)	42(62%)	
Control	108(56%)	–	108(100%)		98(78%)	10(15%)	< 0.001
NH	85(44%)	85(100%)	–		27(22%)	58(85%)	
Any reaction	125(65%)	27(32%)	98(91%)	< 0.001	125(100%)	–	
No reaction	68(35%)	58(68%)	10(9%)		–	68(100%)	
Max severity: 1	70(36%)	21(25%)	49(45%)		70(56%)	–	
Max severity: 2	40(21%)	6(7%)	34(31%)		40(32%)	–	
Max severity: 3	15(8%)	0(0%)	15(14%)		15(12%)	–	
Any systemic	87(46%)	15(18%)	72(67%)	< 0.001	87(70%)	–	
Any local	117(61%)	24(28%)	93(86%)	< 0.001	117(94%)	–	

*NH* nursing home, *IQR* interquartile range

### NH residents reported fewer reactions compared to the control group

**Fig. 1:**
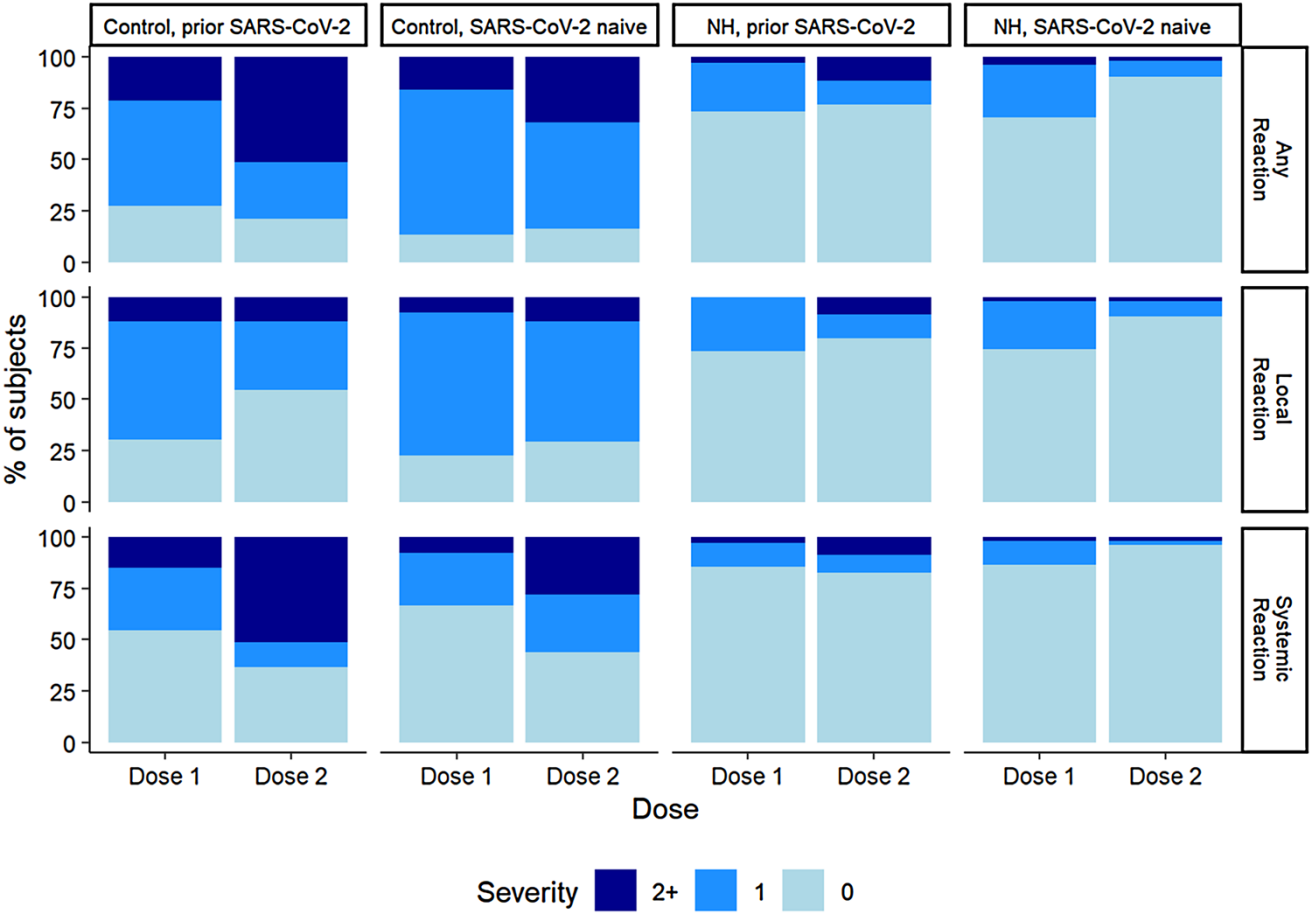
Comparison of reactions reported to the two doses of the BNT162b2 vaccine across different cohorts classified based on a prior infection status. Local reactions include: pain/tenderness, induration, redness, swelling. Systemic reactions include: fever, myalgia, headache, fatigue, rash, shivering, gastrointestinal symptoms (GI, such as nausea, vomiting, diarrhea). Numeric proportion of each strata is contained in Table S1. *NH* nursing home

About two out of every three of our subjects (65%) experienced one or more reactions of varying severity to the vaccine. Over two-thirds of NH residents (68%) did not report *any* reaction to either dose of the vaccine, while most (91%) of the control participants reported some and often more severe symptoms (Fig. 1). None of the NH residents had any grade 3 reactions unlike 14% of the control group (Table 1). While it is believed that females are more likely to report more reactions to vaccines [27], we did not detect a gender difference in the presence or absence of reported reactions (*P* = 0.282).

Although the frequency of symptoms reported for each dose was relatively similar in the total group (59% vs 53%; *p* = 0.091) (Supplemental Table 1), a significantly higher proportion of subjects reported moderate-to-severe reactions to the second dose than the first dose (11% vs 24%; *p* < 0.001) and this increase in moderate-to-severe reactions was observed to some extent in all gender, NH/control, and prior SARS-CoV-2 subgroups. While the younger cohort reported any reaction at the same rate to the first and second doses (82% for both), NH residents reported any reaction significantly more often with the first than the second dose (28% vs. 15%; *p* = 0.015). SARS-CoV-2-naive subjects had symptoms more frequently after the first dose (63% vs. 54%, *p* = 0.014), while prior infected SARS-CoV-2 subjects reported reactions at similar rates to both doses albeit with an increased severity after the second dose (31% vs. 12%, *p* = 0.006). Similarly, women experienced an increase in severity of reactions following the second dose. (See supplemental Table 1 for detailed dose 1 dose 2 group comparisons*).*

### Local injection site and systemic reaction frequency differed by which dose they followed

Local reactions were reported by more subjects than systemic reactions for both doses. While the proportion of subjects reporting local reactions decreased from dose 1 to dose 2 (53–41%), the proportion of subjects reporting systemic reactions increased (27–37%).

**Fig. 2:**
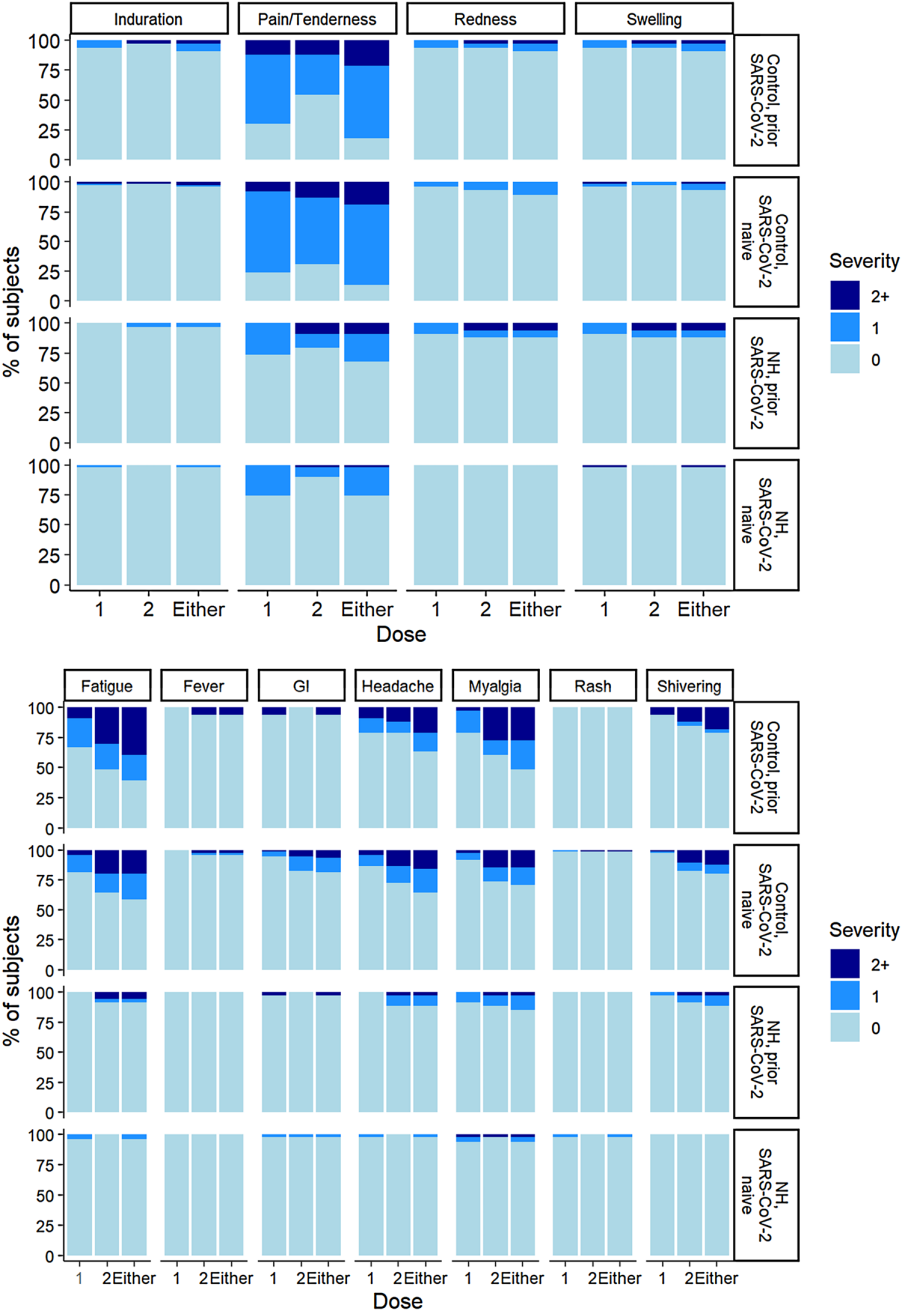
Incidence and severity of local and systemic reactions following administration of two doses of the BNT162b2 mRNA vaccine. Panel A shows the local reactions reported while panel B describes the systemic reactions reported. GI reactions include nausea, vomiting, diarrhea. *NH* nursing home, *GI* gastrointestinal

All groups of subjects reported more local site reactions to the first dose than the second dose of the vaccine (*p* < 0.05) with greater severity among women following receipt of the second dose (*p* = 0.039). Figure 2a displays the percentage of subjects, by prior/naive and NH/control, with specific reported reactions. The most commonly reported local site reaction to both doses (52%) was pain/tenderness, usually described as a “*sore arm.*” Other local site reactions reported include swelling (5%), redness (4%), and less commonly, induration (3%). Following the second dose, systemic reactions occurred with greater frequency and severity than after the first dose (*p* = 0.012 for any reaction; *p* < 0.001 for severe reaction), but this was largely observed in the control cohort. The NH resident cohort, however, did not exhibit such significant disparity in the systemic reactions reported. After the second dose, fatigue and myalgia were the most frequently reported systemic symptoms, accounting for 24 and 20%, respectively. Shivering and headaches were next in frequency, both accounting for 28% of the systemic symptoms reported. Other systemic reactions observed include gastrointestinal symptoms such as nausea, vomiting, and diarrhea, accounting for 7%, followed by 4% who reported fever (Fig. 2b). We did not include the three participants who reported a “mild fever,” because they did not have a temperature recorded.

### Increased reactogenicity correlates with high antibody titers

Tables 2 and 3 show the summary of the antibody responses of each of the four categories of subjects and the antibody levels between those with and without reactions. Overall and in all subgroups presented, subjects with reported reactions have higher GMT antibody levels. Using estimated linear regression models predicting log-transformed anti-spike, anti-RBD, and neutralizing titers, we examined the relationship between reactogenicity and antibody levels. Based on earlier findings in this cohort [24], we adjusted for age, prior SARS-CoV-2 infection, and their interaction in the model in addition to gender and NH/control. To these variables, we added a dichotomous variable indicating the presence or absence of any reported reactions. With this variable, we sought to estimate any difference in immune response between subjects with and without reported reactions after controlling for other predictors of immune response. We found gender and NH/control were not significant predictors in a multivariate model and they were excluded from the final model.

**Table 2:** Antibody response and reactogenicity

	All subjects	NH	Control	NH vs. Control	Reactions	No reactions	Reactions vs. No Reactions
Number of subjects	193	85	108		125	68	
Anti-spike: GMT (95% CI)	4009 (3097, 5190)	2674 (1644, 4347)	5566 (4367, 7093)	0.008	5896 (4757, 7307)	2008 (1119, 3602)	0.001
Anti-RBD: GMT (95% CI)	3629 (2748, 4792)	2232 (1352, 3686)	5378 (4052, 7137)	0.003	5601 (4339, 7231)	1665 (920, 3016)	< 0.001
Neutralizing titer: median (IQR)	411 (161,1254)	230 (63,816)	596 (285,1567)	< 0.001	563 (243,1474)	207 (54,791)	< 0.001
Neutralizing titer: lower limit	10 (5%)	8 (9%)	2 (2%)		2 (2%)	8 (12%)	

*NH* nursing home, *RBD* receptor-binding domain, *GMT* geometric mean titre, *CI* confidence interval, *IQR* interquartile range

**Table 3:** GMT grouped by prior infection and NH/Control

Reaction	SARS-CoV-2-naïve, control		Prior SARS-CoV-2, control		SARS-CoV-2-naïve, NH		Prior SARS-CoV-2, NH	
	Yes	No	Yes	No	Yes	No	Yes	No
Number of Subjects	69	6	29	4	15	36	12	22
Anti-Spike GMT(95% CI)	4533 (3485, 5895)	1798 (175, 18493)	10956 (7220, 16625)	6618 (601, 72937)	2683 (1858, 3874)	776 (321, 1872)	14977 (5867, 38234)	7896 (4201, 14842)
Anti-RBD GMT(95% CI)	4293 (3128, 5891)	1455 (124, 17115)	11375 (6920, 18700)	6891 (415, 114477)	1993 (1188, 3344)	628 (270, 1464)	15887 (5608, 45006)	6578 (3036, 14252)
Neutralizing titer GMT (95% CI)	538 (415, 698)	108 (25, 471)	1642 (823, 3277)	1320 (40, 44061)	156 (91, 267)	111 (61, 204)	1336 (352, 5064)	641 (276, 1489)

*NH* nursing home, *RBD* receptor-binding domain, *GMT* geometric mean titre, *CI* confidence interval

**Fig. 3:**
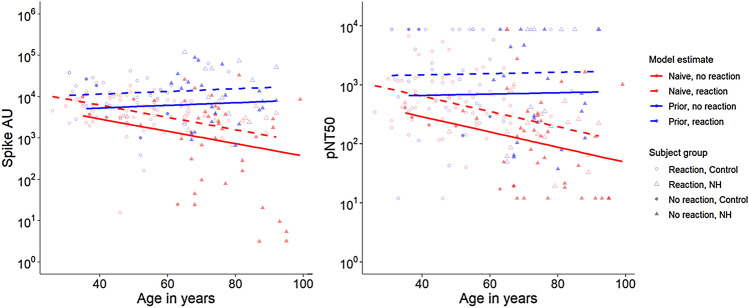
Reactogenicity and prior SARS-CoV-2 infection by antibody response to BNT162b2 mRNA vaccine**.** Panel A shows the anti-spike levels measured across subject age (horizontal axis), NH vs. Control (shape), and reported reaction vs. no reported reaction (shape fill). Overlaid lines depict model-predicted antibody response for those with and without prior SARS-CoV-2 infection (color) and those with and without reported reactions (solid vs. dotted lines). Model estimates reflect lower antibody response observed with increasing age for SARS-CoV-2-naive subjects, but the absence of such a decline in those with prior SARS-CoV-2 infection. After adjusting for age and prior SARS-CoV-2 infection, the differences between antibody response in those with and without reported reactions were statistically significant and are depicted by the distance between the solid and dotted lines. Panel B presents similar findings in neutralizing titers. Additional models comparing antibody response between subjects with no reaction, mild reaction, and moderate/severe reaction did not detect any differences by reaction severity. *AU* arbitrary units, *pNT50* SARS-CoV-2 pseudovirus neutralization titers, *NH* nursing home

In the final model, we observed a statistically significant relationship of the presence of any reaction on immune response, with estimated differences between subjects with and without reactions in log-transformed titer ranging from 0.32 to 0.37 (all *p* < 0.01) (Table S2). This positive difference indicates greater antibody titers in subjects with one or more reported reactions of any severity. In Fig. 3, the lines depict the model-estimated values and illustrate the observed decline in anti-spike titers with age in SARS-CoV-2-naive recipients. This decline in anti-spike with increased age was not present in those with prior infection. Remarkably, higher anti-spike titers were observed in subjects with reported reactions. Models predicting anti-RBD and post-neutralization titers (Fig. 3b, S1) yielded similar overall results and specific estimates of increased response among subjects reporting reactions, suggesting a correlation between reactogenicity and antibody response across our three immunologic measures. Notably, among SARS-CoV-2-naive subjects within the NH cohort, GMT anti-Spike and anti-RBD titers in subjects with reactions were over three times those subjects without reactions (*p* = 0.01 and 0.02 respectively). For each titer, similar effects and estimates were reached using robust regression.

## Discussion

We report a reduced incidence of reactions following BNT162b2 mRNA vaccination in nursing home residents compared to the phase 3 trial in the younger and healthier population. Polack et al., had reported an overall moderate incidence and mild severity of adverse events (AE), both local and systemic, with a lesser frequency and severity in the older cohort (> 65 years) [5]. This aligns with real-world data from other SARS-CoV-2 vaccine studies [18, 19, 28, 29]. While this reduced reactogenicity has largely been attributed to immunosenescence and comorbidities [13–15], tolerance to symptoms may also have a role in reactogenicity in this population. With an increased pain threshold which presumably comes with aging, for instance, pain as a symptom may likely be underreported among this age group. This further underscores the subjectiveness in the report and collation of data on reactogenicity [30]. Unsurprisingly, about 76% of the younger control group in our study reported local injection site pain as the commonest post-vaccination reaction experienced, against a paltry 26% of NH residents. Despite this dramatic disparity between the cohorts, pain at injection sites remains the commonest side effect experienced by these aged vaccinees, especially following the first dose. In line with the clinical trials and real-world studies, transient local site reactions were more frequent than systemic side effects which could be more worrisome to vaccinees [31, 32].

In contrast to local reactions, like others [28], we elicited more systemic reactions with the second dose independent of prior infection status. An antigenic priming of the immune system, as would be anticipated from previous infection or the first vaccine dose, could inadvertently translate to increased reactogenicity following subsequent antigenic exposure via a booster by vaccine or even re-infection. This could explain the increased systemic reactogenicity and immunogenicity observed with the second dose in most of our participants, as well as that reported by others [18, 19]. While this was particularly true about our younger community-dwelling subjects, the older NH residents had little difference in systemic reactions between the two doses, further highlighting the blunting of their immune response.

Given the favored notion that increased reactogenicity could result from increased immunogenicity, few have reported their exploration of this relationship. In a study comparing the induction of immune response between young and elderly cohorts of SARS-CoV-2-naive subjects, Muller et al. observed a negative correlation of antibody response and age, but did not observe any significant correlation between post-vaccination reactions and antibody response to the BNT162b2 mRNA vaccine [33]. Systematic differences in soliciting for reactions or an artifact of population differences due to geographical differences in how adverse events are reported may have caused the disparity from our observations [34–36]. Additionally, our observed association between reactogenicity and immune response was adjusted for age and prior SARS-CoV-2 infection which differs from the analysis described by Muller. In our model, we observed that SARS-CoV-2-naive subjects had reduced antibody titers with increasing age unlike subjects who had a prior infection. This agrees with studies suggesting that prior SARS-CoV-2 infection may blunt the age-dependent decline that has been noted in immunogenicity to vaccines [24, 37]. Moreso, testing reactogenicity as a predictor of log-transformed anti-spike, anti-RBD, and functional neutralizing titers, our model revealed a statistical significance in all three immune measures indicating higher titers, and consequently increased antibody production, in subjects who reported reactions to the vaccine. This model prediction may have clinical implications. It suggests that reactogenicity correlates directly with immunogenicity, i.e. in the presence of any reaction, even at least one mild reaction, one may predict a correspondingly greater antibody response.

The observations reported in this study have limitations. As the vaccine has only been in use for a few months, only short-term reactions are identified and reported. Continued surveillance is necessary to monitor possible effects that may ensue long-term. Moreso, due to the small size of our study population, we were underpowered to assess specific effects within the respective control and NH cohorts. Large-scale studies are needed to further confirm the findings reported in this study. In addition, comorbidities, which may contribute to reduced immune response, were not considered in this study. Finally, different measures of subjectiveness affect the report of reactions by subjects in general, even as the search for a more objective and less biased method of soliciting vaccine side effects continues. By conducting interviews at the point of reactogenicity log retrieval, we attempted to limit a recall bias usually associated with self-reported surveys. Nevertheless, fever as well as induration remain a subjective assessment in the absence of a thermometer and ruler, respectively, which may have affected the frequencies and severity of those reactions as included in our dataset.

This study has implications for the tolerance of mRNA vaccination in NHs. We show that the BNT162b2 mRNA vaccine is well tolerated among NH residents with a much milder reactogenicity profile than reported for subjects within a similar age group in the clinical trials. Our study suggests that the presence of reactogenicity could serve as a proxy for predicting immunogenicity to the BNT162b2 mRNA vaccine. While we advocate for confirming our observation through larger scale studies, our hypothesis-generating observation may have clinical usefulness in screening and targeting booster doses among vaccinated older adults. It also provides positive framing of reactogenicity when advocating for vaccine acceptance. Should our observation be confirmed to be useful, clinicians will need to change practice and note for those vulnerable subjects that have no reactions for consideration of future booster. Also, for those with tepid vaccine acceptance, it could serve to somewhat mitigate concerns about vaccine reactions as an encouraging sign of vaccine effectiveness.

## Supplementary Information

Below is the link to the electronic supplementary material.

Supplementary file1 (DOCX 95 kb)

## Data Availability

All data generated or analyzed during this study are included in this published article [and its supplementary information files]. Additional datasets generated during and/or analyzed during the current study are available from the corresponding author on reasonable request.
