# Supplementary material for: Does a lack of vaccine side effects correlate with reduced BNT162b2 mRNA vaccine response among healthcare workers and nursing home residents?
Source: Aging Clin Exp Res. 2021 Oct 15;33(11):3151–60. doi: 10.1007/s40520-021-01987-9 (PMC8518269; doi:10.1007/s40520-021-01987-9)
Supplement: Supplementary file 1 — Supplementary file1 (DOCX 95 kb) [file 40520_2021_1987_MOESM1_ESM.docx]

**Supplement**

Table SⅠ

| **Reactions** | **Subgroup** | **Dose 1** | **Dose 2** | **p-value** | **Dose 1**  **Moderate/ Severe** | **Dose 2**  **Moderate/ Severe** | **p-value** |
| --- | --- | --- | --- | --- | --- | --- | --- |
| **Any reactions** | **Overall** | 113 (59%) | 102(53%) | 0.091 | 22 (11%) | 46 (24%) | 0.000 |
|  | **Control** | 89 (82%) | 89 (82%) | 1.000 | 19 (18%) | 41 (38%) | 0.001 |
|  | **NH** | 24 (28%) | 13 (15%) | 0.015 | 3 (4%) | 5 (6%) | 0.617 |
|  | **Female** | 59 (65%) | 56 (62%) | 0.546 | 14 (15%) | 33 (36%) | 0.000 |
|  | **Male** | 54 (53%) | 46 (45%) | 0.153 | 8 (8%) | 13 (13%) | 0.332 |
|  | **Naive** | 80 (63%) | 68 (54%) | 0.014 | 14 (11%) | 25 (20%) | 0.037 |
|  | **Prior** | 33 (49%) | 34 (51%) | 1.000 | 8 (12%) | 21 (31%) | 0.006 |
| **Local reactions** | **Overall** | 103 (53%) | 80 (41%) | 0.002 | 11 (6%) | 17 (9%) | 0.286 |
|  | **Control** | 81 (75%) | 68 (63%) | 0.049 | 10 (9%) | 13 (12%) | 0.628 |
|  | **NH** | 22 (26%) | 12 (14%) | 0.016 | 1 (1%) | 4 (5%) | 0.371 |
|  | **Female** | 54 (59%) | 43 (47%) | 0.037 | 5 (5%) | 15 (16%) | 0.024 |
|  | **Male** | 49 (48%) | 37 (36%) | 0.038 | 6 (6%) | 2 (2%) | 0.221 |
|  | **Naive** | 71 (56%) | 58 (46%) | 0.026 | 7 (6%) | 10 (8%) | 0.579 |
|  | **Prior** | 32 (48%) | 22 (33%) | 0.055 | 4 (6%) | 7 (10%) | 0.505 |
| **Systemic reactions** | **Overall** | 52 (27%) | 71 (37%) | 0.012 | 13 (7%) | 42 (22%) | 0.000 |
|  | **Control** | 40 (37%) | 63 (58%) | 0.001 | 11 (10%) | 38 (35%) | 0.000 |
|  | **NH** | 12 (14%) | 8 (9%) | 0.343 | 2 (2%) | 4 (5%) | 0.480 |
|  | **Female** | 36 (40%) | 42 (46%) | 0.286 | 10 (11%) | 30 (33%) | 0.000 |
|  | **Male** | 16 (16%) | 29 (28%) | 0.026 | 3 (3%) | 12 (12%) | 0.027 |
|  | **Naive** | 32 (25%) | 44 (35%) | 0.059 | 7 (6%) | 22 (17%) | 0.001 |
|  | **Prior** | 20 (30%) | 27 (40%) | 0.146 | 6 (9%) | 20 (30%) | 0.004 |

Table SⅡ

|  | **Spike** | | **RBD** | | **Neutralizing titer** | |
| --- | --- | --- | --- | --- | --- | --- |
| **Model variable** | **Coefficient** | **p-value** | **Coefficient** | **p-value** | **Coefficient** | **p-value** |
| **Intercept** | 3.311 | <0.001 | 3.248 | <0.001 | 2.357 | <0.001 |
| **Prior COVID vs. Naive** | 0.45 | <0.001 | 0.475 | <0.001 | 0.5 | <0.001 |
| **Age (years, centered at 50)** | -0.015 | <0.001 | -0.017 | <0.001 | -0.014 | <0.001 |
| **Prior COVID * Age** | 0.018 | 0.003 | 0.02 | 0.002 | 0.015 | 0.013 |
| **Any reaction** | 0.335 | 0.004 | 0.372 | 0.003 | 0.321 | 0.005 |

**Fig. SⅠ**

**
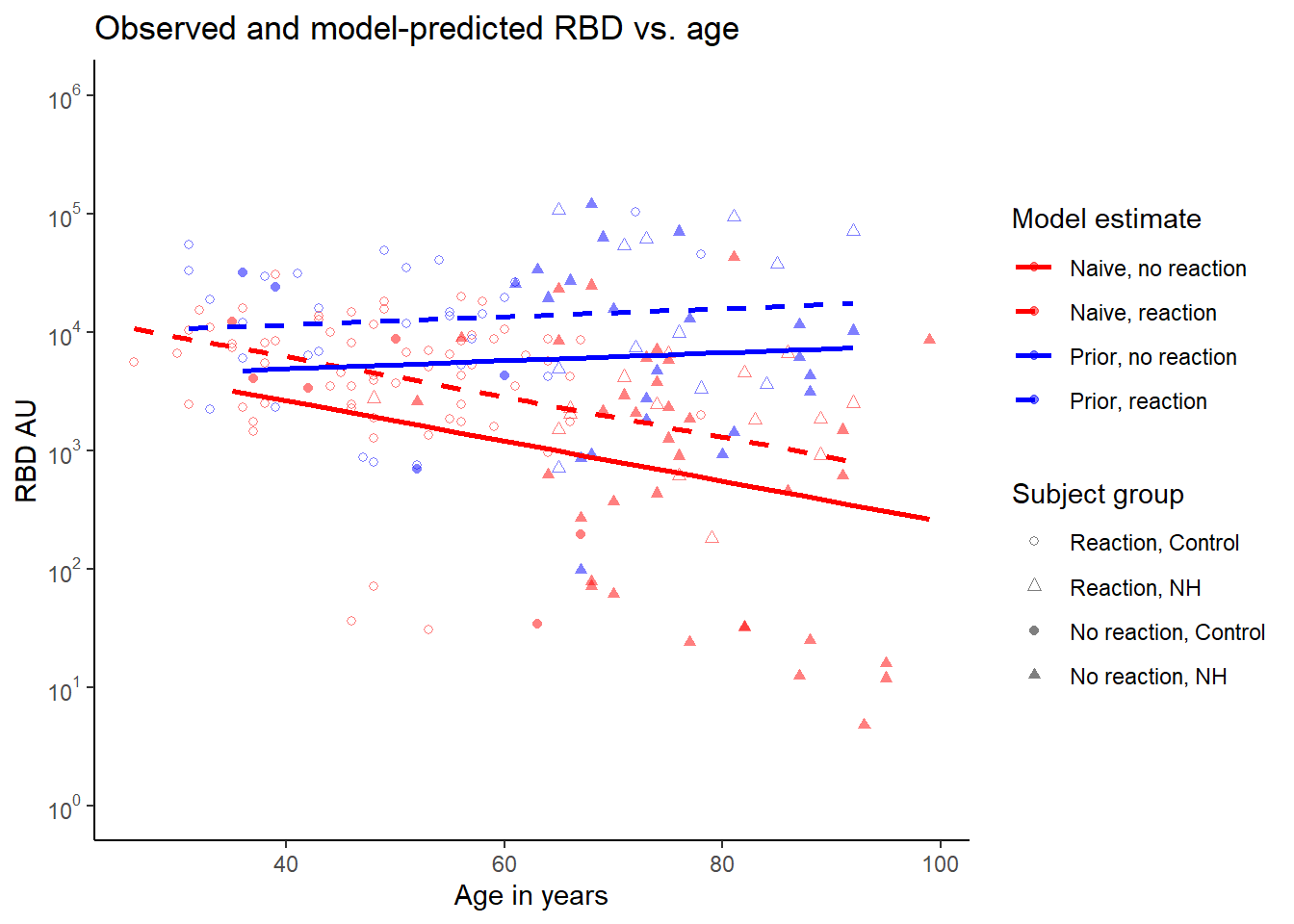
 Figure S1. Reactogenicity and prior SARS-CoV-2 infection by antibody response to BNT162b2 mRNA vaccine**

Fig. S1 shows the anti-RBD levels measured across subject age (horizontal axis), NH vs. Control (shape), and reported reaction vs. no reported reaction (shape fill). Overlaid lines depict model-predicted antibody response for those with and without prior SARS-CoV-2 infection (color) and those with and without reported reactions (solid vs. dotted lines). Model estimates reflect lower antibody response observed with increasing age for SARS-CoV-2 naive subjects, but the absence of such a decline in those with prior SARS-CoV-2 infection. After adjusting for age and prior SARS-CoV-2 infection, the differences between antibody response in those with and without reported reactions were statistically significant and are depicted by the distance between the solid and dotted lines. Abbreviations: AU, arbitrary units; pNT50, SARS-CoV-2 pseudovirus neutralization titers; NH, nursing home
